# Response to Comments on Montagna et al. “Evaluation of Legionella Air Contamination in Healthcare Facilities by Different Sampling Methods: An Italian Multicenter Study” *Int. J. Environ. Res. Public Health* 2017, *14*, 670

**DOI:** 10.3390/ijerph14080906

**Published:** 2017-08-11

**Authors:** Maria Teresa Montagna, Osvalda De Giglio, Antonella Agodi, Cesira Pasquarella

**Affiliations:** 1 Department of Biomedical Science and Human Oncology, University of Bari “Aldo Moro”, Piazza G. Cesare 11, 70124 Bari, Italy; osvalda.degiglio@uniba.it; 2 Department of Medical and Surgical Sciences and Advanced Technologies “GF Ingrassia”, University of Catania, Via Sofia 87, 95123 Catania, Italy; agodia@unict.it; 3 Department of Medicine and Surgery, University of Parma, Via Volturno 39, 43125 Parma, Italy; ira.pasquarella@unipr.it

We would like to thank Collins and Walker for their comments and for acknowledging that this is an area requiring more research to improve our basic understanding of *Legionella* [1]. We agree with Collins and Walker’s suggestion that some characteristics of tap outlets and the bathrooms can influence aerosols produced and may facilitate interpretation of the aerosol data. However, this information was not taken, not being in the aims of the study. These aspects could certainly be considered in future studies, to characterize the aerosols produced by the outlets, using, for example, an aerodynamic particle sizer (APS) [2].

Regarding the viable air-borne *Legionella*, frequently detected at low concentrations (i.e., 1 cfu/m^3^ of air or 1 cfu per hour of sampling), our conclusions from the culture data are based on the presence/absence of *Legionella*, not on concentration data. Furthermore, to confirm the contamination source, *Legionella pneumophila* strains isolated from air and water samples were compared by SBT (sequence-based typing). Results let us identify the same SB type from three out of four healthcare facilities which tested positive for *Legionella* in the air by at least one culture-based method [3].

The article of Wiik et al. [4] reports that a high concentration of *Legionella* (>300,000 cfu/L) in water is required to detect *Legionella* in the air. We apologize for not having mentioned this paper, but our *Legionella* data range in water samples agrees with results reported by Wiik et al. [4]; that is, viable air-borne *Legionella* was infrequently detected at low concentrations in water samples.

We agree with Collins and Walker’s suggestion about the need for future investigations regarding genomic unit (GU) studies. We are going to plan a comparison between the number of GUs in the water with the number of GUs in the air via molecular analysis by real-time PCR (polymerase chain reaction) to give a better “estimation” of the emission factor.

We agree about the several key unanswered questions [1] which should be research priorities considering a globally increasing incidence of Legionnaires’ disease. Regarding the first question on what forms of *Legionella* are present in aerosols (*viable* or *not viable*) [1], we think that in the legionellosis risk analysis it is important to evaluate the presence of *Legionella* regardless of *viable* and *not viable*. However, we are aware that distinguishing the *viable* and *not viable* forms of the microorganism is very important to reduce the overestimation of the risk in case of epidemiological inquiry. As we reported in our manuscript [3], the molecular-based method real-time PCR could be combined with measurements of viability such us DNA pre-treatment with ethidium monoazide (EMA) viable dye and propidium monoazide (PMA). In our study, this DNA pre-treatment was lacking, but this could be an object of future research to add new information to deepen our knowledge on this issue.

We would like to thank Collins and Walker for their positive criticism and for any possible future collaboration. Moreover, we thank the Editor for giving us the opportunity to provide a reply to the letter.
